# Distributed Pedestrian Detection Alerts Based on Data Fusion with Accurate Localization

**DOI:** 10.3390/s130911687

**Published:** 2013-09-04

**Authors:** Fernando García, Felipe Jiménez, José Javier Anaya, José María Armingol, José Eugenio Naranjo, Arturo de la Escalera

**Affiliations:** 1 Intelligent Systems Laboratory, Universidad Carlos III de Madrid, Avda de la Universidad 30, Leganés 28911, Madrid, Spain; E-Mails: armingol@ing.uc3m.es (J.M.A.); escalera@ing.uc3m.es (A.D.L.E.); 2 INSIA, Technical University of Madrid, Carretera de Valencia, km.7, Madrid 28031, Spain; E-Mails: felipe.jimenez@upm.es (F.J.); jj.anaya@upm.es (J.J.A.); joseeugenio.naranjo@upm.es (J.E.N.)

**Keywords:** Advance Driver Assistance Systems, VANETs, V2V, data fusion, laser scanner and computer vision

## Abstract

Among Advanced Driver Assistance Systems (ADAS) pedestrian detection is a common issue due to the vulnerability of pedestrians in the event of accidents. In the present work, a novel approach for pedestrian detection based on data fusion is presented. Data fusion helps to overcome the limitations inherent to each detection system (computer vision and laser scanner) and provides accurate and trustable tracking of any pedestrian movement. The application is complemented by an efficient communication protocol, able to alert vehicles in the surroundings by a fast and reliable communication. The combination of a powerful location, based on a GPS with inertial measurement, and accurate obstacle localization based on data fusion has allowed locating the detected pedestrians with high accuracy. Tests proved the viability of the detection system and the efficiency of the communication, even at long distances. By the use of the alert communication, dangerous situations such as occlusions or misdetections can be avoided.

## 1. Introduction

Of all the problems that are related to transportation, traffic accidents are the most dramatic since they deal with human lives. The efforts of recent years, such as an increase in the safety measures for roads and vehicles or the enhancement of traffic laws to decrease drivers&apos misbehaviors, have led to reduced death tolls in road accidents, yet each year in the European Union more than 1 million road accidents still occur in which over 31 thousand people die [1]. Thus, even though the efforts made are helping to mitigate this number, there is still a considerable amount of work to be done. The new information and communication technologies developed in the last decade have enabled more complex and reliable safety applications to be created. These new applications are able to reduce the number of accidents and deaths on the road by both preventing them and abating the harm caused by accidents. Most traffic accidents are related to human errors. Carelessness and erroneous decisions by the driver are the two main factors that cause traffic accidents. These kinds of errors, related with human nature, are impossible to be eliminated, although efforts can be made to decrease them. Recent research in the field of Intelligent Vehicles (IV) has focused on using advances in information technologies to prevent these errors. Advanced Driver Assistance Systems (ADAS) try to warn the driver and prepare the driver in the event of hazardous situations. During recent years, Intelligent Transportation Systems (ITS) research projects have been abundant in Spain, with several laboratories working in different fields related to the topic at hand. INSIA and Universidad Complutense de Madrid have presented different works related to the time to collision calculation for vehicle intersections [2] and autonomous maneuvers for vehicle avoidance [3]. On the other hand, Carlos III University has proposed several works related with sensor detection classification and tracking [4]. In recent years, these works, sponsored by the Spanish government, have led to the presentation of several works on the topic [5,6]. Other laboratories in Spain are also working on the topic with important contributions, such as the works presented by Universitat Autònoma de Barcelona [7,8] and Universidad de Alcalá de Henares [9,10].

The work presented in this paper represents a step forward in pedestrian detection and danger communication. The use of Data Fusion techniques to enhance the capabilities of classic Advanced Driver Assistance Systems allows improving the performance of the detection system, based on the use of two well-known solutions, laser scanners and computer vision. Furthermore, the communication system allows providing accurate and fast pedestrian detection based on the precise location of the vehicle and the use of a very accurate obstacle detection device, *i.e*, a laser scanner. The system is based on the occlusion scenario, where pedestrians are occluded by other vehicles on the road. This situation is frequent in urban environments and represents one of the most typical accidents involving pedestrians. The proposed solution consists of the fact that the vehicle that occludes a pedestrian automatically informs other vehicles in the surroundings of the presence of the pedestrian by means of a pedestrian detection and alert communication protocol. In these situations, the availability of accurate sensors, able to detect pedestrians with robustness, is as important as the capability to inform the vehicles in the surroundings, based on trustable technology. The communication to the vehicles has to be fast and reliable and able to adapt to fast changing scenarios such as urban environments.

Among the different contributions presented in this paper, we can highlight an improved laser scanner-based pedestrian detection system, the high level fusion system that combines information from a laser scanner and artificial vision, together with the tracking system, and the novel track definition (consolidated/non-consolidated).

## 2. Related Work

Data Fusion is becoming frequent in ITS works, due to the need for reliable and trustable sensor systems for road safety applications. The fusion of different sensing devices helps to overcome the limitation of each sensing technology, providing accurate and trustable detections.

Fusion approaches in vehicle safety are divided according to the fusion architecture used. Most of these works focus on the classification process:

In *decentralized* approaches, detections and classifications are performed by an independent subsystem with limited information (typically a single sensor). A final stage combines the information and classification, according to the sensors and the certainty of the detections. In [11], Adaboost vision-based pedestrian detection and Gaussian Mixture Model classifier (GMM) for laser scanner-based pedestrian detections, a Bayesian decisor is used to combine detections of both subsystems. In [12], multidimensional features for laser scanner pedestrian detection are used, and Histograms of Oriented Gradients (HOG) features and Support Vector Matching (SVM) for visual detection is performed by a Bayesian model approach. In [13], a similar approach is presented in comparison with other medium level algorithms.

In *centralized* architectures, a set of combined features from different sensors is created. Hence a single classification stage is necessary with the information from all the available sensors. A classical centralized data fusion approach for pedestrian detection is stereo vision systems, although some authors tend to consider stereo vision as a single scanner. Other works take advantage of different sensing technologies: In [13,14], a complete work with tests of different algorithms is presented to combine the features from different sensing devices, again based on laser scanner and computer vision. The alternative methods presented are Naïve Bayes, GMMC, NN, FLDA, and SVM. The work in [15] uses feature level information to improve the tracking of the objects.

Other approaches take advantage of the trustability of the laser scanner to detect the Region of Interest (ROI) in an image and perform computer vision detection taking advantage of the amount of information available from the computer vision sensor. In [16] SVM is used for vision-based vehicle detection and classification, while [17] uses Convolutional Neural Networks for pedestrian detection and [18] HOG and SVM approach. Finally, [19] uses Invariant Features and SVM to perform vision-based pedestrian detections. Based on a different point of view, the authors of [20] search in the environment for dangerous zones where pedestrians could be located, based on the laser scanner information (e.g., space between two vehicles). In those regions, pedestrian classification is performed based on a vision approach. Although these approaches deal with several sensors, each process (classification and detection) is performed by a single sensor, thus the fusion process is limited to the data alignment process.

It is clear that the information retrieved by a vehicle provided only by its local sensors should be enough to prevent near accidents or to reduce the effects of a certain accident. However, new vehicle applications such as Cooperative Collision Warning [21], Cross-Flow Turn Assistant [22], Curve Speed Warning [23], Emergency Vehicle Warning [24] and others, require additional information on the circulation environment, from the other vehicles as well as from the infrastructure. This means that an external source of information is necessary in the vehicle itself in order to provide the necessary information to guarantee a proper performance of these assistance systems. Vehicle to Vehicle (V2V) and Vehicle to Infrastructure (Roadside) (V2I) communications cover the gap of providing information to the Advanced Driver Assistance Systems (ADAS) from the surroundings of a vehicle.

## 3. General Description

When dealing with safety applications, a fast and reliable obstacle identification and report method is required. But perception sensors have limitations due to the technology used (e.g., range limitation, usability, occlusions). The combination of different sensors can overcome these problems. But urban scenarios are fast-changing and unstructured. Thus, situations arise where detections are unfeasible. A distributed pedestrian detection can help to detect a pedestrian even in the most challenging scenarios, allowing the vehicle to perceive information that would be unattainable in a normal situation. This distributed information can be provided by other vehicles or the infrastructure.

The work at hand focuses on a distributed detection and alert of pedestrians. A fusion approach is used for reliable pedestrian detection and localization, and a trustable communication protocol alerts other vehicles of the situation in advance. In the fusion architecture created, the Kalman Filter (KF) approach is included for pedestrian tracking. The Kalman Filter represents an important part of the approach allowing us to track pedestrians and to fuse the information of the detection of both sensors, as explained in Section 4.

**Figure 1.**
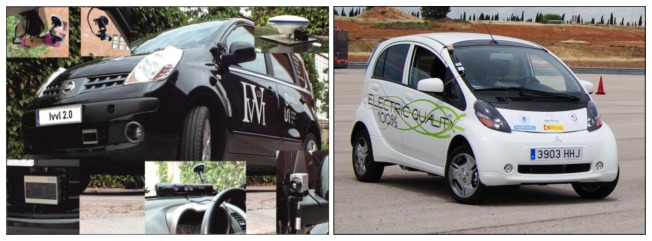
Left platform IVVI 2.0 with all the sensing devices available. Right vehicle with the communication receiver.

Two vehicles were used (Figure 1). The first one is a research platform for ADAS test and development, with multiple sensing devices incorporated. The second platform is a vehicle equipped with an on-board computer able to display the detection to the driver. In the present application three sensing devices where mainly used, an accurate high definition GPS with inertial measurement, used for accurate localization of the vehicle and the pedestrians, and two sensors used for pedestrian detection (laser scanner and computer vision). Both vehicles were equipped with the communication devices (MTM-CM3100 gateways, Maxfor Inc., Santa Clara, CA, USA) that provided the communication channel vehicles.

The work represents a step forward, combining a state-of-the-art pedestrian detection algorithm with the capacity of Vehicle to Vehicle communications (V2V), creating a robust pedestrian detection and alert communication.

## 4. Data Fusion Based Pedestrian Detection

As previously mentioned, the pedestrian detection approach presented is based on the use of two well-known technologies in Intelligent Transportation Systems, a laser scanner and computer vision. The former is a recent sensor in automotive applications that helps in the process of creating intelligent applications thanks to its trustworthiness and reliability, but the information provided is limited. On the other hand computer vision provides a high amount of information. This information is unstructured, and with limited reliability. The combination of both systems helps to overcome the limitations inherent to each system resulting in a reliable application that fulfills the key requirements of safety applications.

In this section, a pedestrian detection system is described for each subsystem (laser scanner and computer vision). Also, procedures to extrapolate laser scanner detections to the computer vision coordinate system are specified; finally, all these detections must be extrapolated to the vehicle coordination system that is the front part of the vehicle. Using a high precision GPS with an inertial measurement system (MTI-G) from Xsens Technologies B.V. (Enschede, The Netherlands), it is possible to obtain precise localization of the vehicle and thus of the pedestrians around. All this information is provided to the second vehicle, obtaining accurate localization of the pedestrians detected.

### 4.1. Laser Scanner Pedestrian Detection

The laser scanner was mounted in the bumper of the test platform IVVI 2.0 (Figure 1). Before pedestrian classification, obstacle segmentation is mandatory, with shape estimation. Later, this information is used to select which of the detected obstacles are suitable to be classified as pedestrians. Later, obstacle classification is performed taking into account the previously estimated shape.

#### Obstacle Segmentation and Shape Estimation

**Figure 2.**
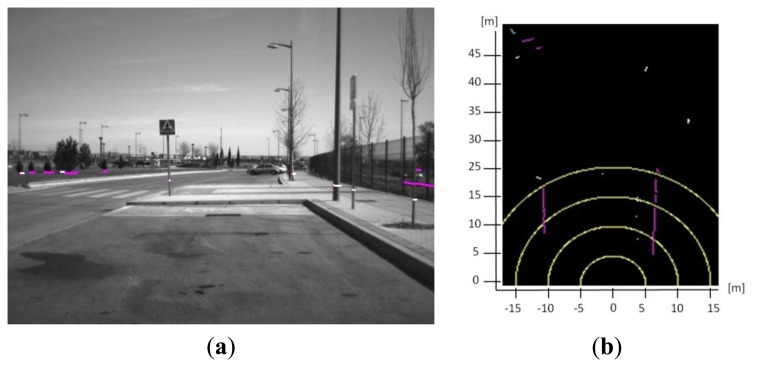
Laser scanner clustering and shape reconstruction. (**a**) The points are shown in the image field; (**b**) the information only from the laser scanner is shown with the polylines reconstruction, where yellow lines represent the distances 5, 10, 15 and 20 m to the laser scanner.

Laser scanner rotation provides 401 distance points per scan according to the rotation angle with 0.25 degrees of resolution, each one of them is provided with a time delay with respect to the others. Thus, movement of the vehicle has to be compensated using the information given by the high precision GPS system. Euler angles displacement and velocity must be taken into account in order to provide accurate shape reconstruction. Furthermore, the laser scanner is also very sensitive to pitching movements, so this information is also used to check if there is a strong pitch movement that can lead to misdetections. Applying the compensation with the rotation and translation matrixes (Equation (1)) the points are referenced to the position of the last point received (Figure 2).

(1) $$\begin{array}{c} \left[\begin{array}{c} x\\ y\\ z \end{array}\right]=R(\left[\begin{array}{c} x_{0}\\ y_{0}\\ z_{0} \end{array}\right]+T+T_{0})\\ R=\left[\begin{array}{ccc} \cos(\Delta \delta ) & 0 & \sin(\Delta \delta )\\ 0 & 1 & 0\\ -\sin(\Delta \delta ) & 0 & \cos(\Delta \delta ) \end{array}\right]\;\left[\begin{array}{ccc} 1 & 0 & 1\\ 0 & \cos(\Delta \varphi ) & -\sin(\Delta \varphi )\\ 0 & \sin(\Delta \varphi ) & \cos(\Delta \varphi ) \end{array}\right]\;\left[\begin{array}{ccc} \cos\left(\Delta \theta \right) & -\sin\left(\Delta \theta \right) & 0\\ \sin(\Delta \theta ) & \cos(\Delta \theta ) & 0\\ 0 & 0 & 1 \end{array}\right]\\ T_{0}=\left[\begin{array}{c} vT_{i}\times \cos\left(\Delta \varphi \right)\\ vT_{i}\times \sin\left(\Delta \varphi \right)\\ 0 \end{array}\right],T_{0}=\left[\begin{array}{c} x_{t}\\ y_{t}\\ z_{t} \end{array}\right] \end{array}$$

In Equation (1), ΔΔ, Δφ, and Δ*θ* correspond to the increment of the Euler angles roll, pitch and yaw respectively for a given period of time *T_i_*. Coordinates (x,y,z) and (x_0_,y_0_,z_0_) are the Cartesian coordinates of a given point after the vehicle movement compensation. *R* is the rotation matrix, *T_v_* the translation matrix according to the velocity of the vehicle, *T*_0_ the translation matrix according to the position of the laser and the inertial sensor. Finally, after clustering the shapes of the different obstacles are estimated using polylines. Polyline creation as well as detailed explanation of the clustering algorithm is given in [25] (Figure 2).

#### Obstacle Classification

After shape estimation, classification is performed, differentiating among the following kind of obstacles: Big obstacles, Road limits, Vehicles, L shaped, Small obstacles and Pedestrians. Present applications focus on pedestrian detection, and a detailed explanation of the other types of obstacles is provided in [6].

**Figure 3.**
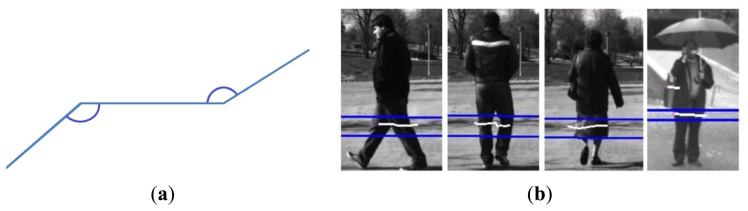
(**a**) Pattern for pedestrian detection. (**b**) Different examples of different patterns given by pedestrians with different leg positions. The blue lines represent the boxes including pedestrian detections of the laser scanner.

Final pedestrian classification is performed in three steps, using *a priori* contextual information. Firstly, obstacles with a size proportional to a pedestrian are selected, based on anthropometric studies among the different obstacles; later, the shape of the polyline is checked with a typical pedestrian pattern; finally, a tracking stage is included, using the anthropometric information to avoid false positives. The idea behind this method was presented for the first time in [25], although the high rate of false positives obtained required a redesign of certain aspects, *i.e.*, tracking stage to avoid false positives or the use of context information based on anthropometric studies. A study of the different patterns given by pedestrians was performed giving the pattern shown in Figure 3a.

#### Pattern Matching

In this pattern three polylines are presented and the angles that connect the polylines are included within the limits of $\left[0,\frac{\pi }{2}\right]$.

Pattern matching process computes the two angles and gives a similarity score:

(2) $$\text{Similarity}=\frac{2\theta_{1}}{\pi }\times \frac{2\theta_{2}}{\pi }$$

where *θ*_1_ and *θ*_2_ are the angles that connect two consecutive lines.

This similarity is computed among consecutive polylines that fit with the pedestrians' size. If the result is greater than a given threshold, the obstacle is considered to be a pedestrian. It is assumed that a previous pattern is common in unstructured scenarios, thus false positives are expected. Using information fusion with computer vision, this limitation can be overcome.

The specified pattern proved to be a useful tool, even in scenarios with certain difficulties, such as pedestrians wearing skirts or static pedestrians, where the detection of pedestrians using the specified pattern represents a higher challenge. As shown in Figure 3b, the results provided by the pattern in these specific scenarios were very positive. The similarity threshold was empirically chosen after the study of several sequences in controlled scenarios, selecting the threshold that assured positive rates over 80% in all movements and static pedestrians.

Information provided by the laser scanner is limited, due to the specific nature of the sensor, to a 401 distance. Thus, providing a trustable classification is a relatively difficult task. In order to avoid misdetection, a higher level stage was designed to avoid errors in the classification process. This stage consisted of the estimation of the movement based on KF, and feature-based correlation (features). This tracking stage helps to study the movement of the pedestrians, eliminating those pedestrians that do not fit with the movement of a pedestrian (e.g., fast speeds or size changes). Finally, there is an upper level classification based on a voting scheme, according to the classification in the last ten scans:

$$\text{Features}:<\bar{N},\text{height},\text{width},\delta_{+x},\delta_{-x},\sigma ,d,\rho >$$

Obstacle features used for comparison, where N̅ is the medium number of points, height and width are the sizes of the obstacle, σ is the standard deviation of the points to the center of the obstacle. *ρ* is the radius of the circle that surrounds the obstacle and d is the distance to the estimation of the KF. Finally δ_+x_, δ_-x_ are the numbers of points to the left or to the right of the center. Before ROI detection some data alignment must be performed since sensors do not share the same coordination system. This data alignment is explained in the following section.

### 4.2. Vision Based Pedestrian Detection

**Figure 4.**
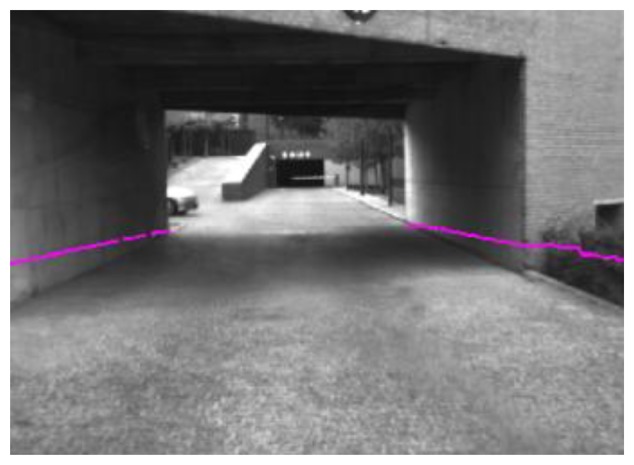
Laser scanner detection extrapolated to the camera field of view with accurate extrinsic calibration.

Laser scanner detections are extrapolated to the field of view of the camera, thus only obstacles given by the laser scanner and extrapolated to the image are processed to check whether they are pedestrians or not. This way, the reliability of the laser scanner is used to reduce the false positives provided by the vision systems. In addition, the amount of information used for visual processing is reduced by reducing the region of the images processed by the computer vision system to those provided by the laser scanner (Figure 4), allowing real time processing. If the laser scanner is unavailable (e.g., strong pitch movement), the whole image is used to process the pedestrian detection.

To provide the laser scanner information to the camera coordination system a pin-hole model was used together with a high-accuracy extrinsic calibration system. Both coordination frames of the two different subsystems were translated to the reference point, which is the central point of the front bumper of the vehicle. The calibration process was performed online, allowing recalibration in real time of the Euler angles in case of necessity. The calibration process is performed online and supervised. Once the equipment is mounted and the laser scanner detection points are displayed on the image, they are varied online until they match with the real image. Supervised online calibration is a process performed taking into account the extreme sensitivity of the laser scanner to pitch angle.

To perform this coordinate change, transformation and rotation matrix must be used:

(3) $$\left[\begin{array}{c} x_{v}\\ y_{v}\\ z_{v} \end{array}\right]=R_{i}(\left[\begin{array}{c} x_{i}\\ y_{i}\\ z_{i} \end{array}\right]+T_{i})$$

where *T_i_* is the translation matrix and *R_i_* is the rotation. This rotation matrix is equivalent to the rotation matrix used in Equation (1). but in this case, Euler angles correspond to the angular deviation among the coordinate system. *T_i_* is equivalent to the displacement in the Cartesian coordinate system for each sensor:

(4) $$\lambda \left[\begin{array}{c} u\\ v\\ 1 \end{array}\right]=\left[\begin{array}{ccc} f & 0 & u_{0}\\ 0 & f & v_{0}\\ 0 & 0 & 1 \end{array}\right]\;\left[\begin{array}{c} x_{c}\\ z_{c}\\ y_{c} \end{array}\right]$$

where *u*, *v* are the image coordinates in pixels *f* the focal distance and (*x_c_*,*y_c_*,*z_c_*) the Cartesian coordinates of the image detections in the image coordinate system. *u*_0_ and *v*_0_ are the coordinates of the center of the image.

**Figure 5.**
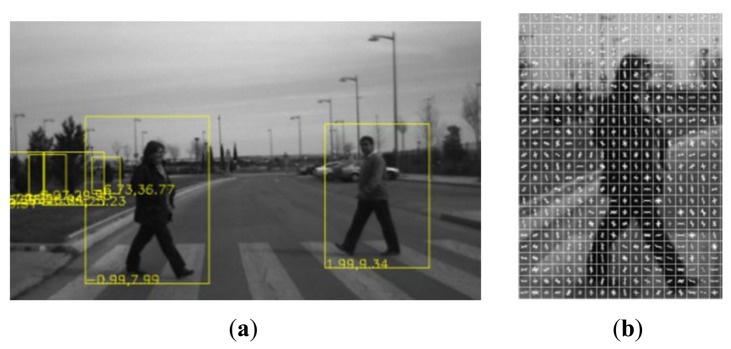
Laser scanner ROI detection and HOG features computed in one of the regions. Figure (**a**) shows the boxes that represent the ROIs given by the laser scanner with the distances highlighted; (**b**) shows the computation of the HOG features in one of these ROIs.

Equations (3) and (4) were also used to transform laser scanner obstacles that fit with pedestrian size to camera coordinate system. Providing Regions of Interest (ROIs) where the classification is performed. This way, obstacle association from both sensors is implicit, since they perform classification for each sensor over the same set of obstacles provided by the laser scanner. This way accuracy and trustability of the laser scanner is used to provide trustable and accurate obstacle detection (Figure 5).

Vision based pedestrian detection is based on the Histogram of Oriented Gradients (HOG) descriptor and Support Vector Machine (SVM) classification [26]. The theory behind the HOG features description is based on local appearance and shape of all objects in an image, which can be described by the distribution of intensity gradients or edge directions. The implementation divides the image into small-connected regions (cells) that can have different shapes (circles or squares). For each cell, a histogram of gradient directions (or edge orientations) for the pixels within the cell is compiled. These histograms are later weighted according to the magnitude of the gradients of the computed cells, and later normalized according to blocks of cells. These blocks of cells can have different shapes and can overlap, thus a given cell can be included in more than one block. The combinations of all these histograms representing the occurrence of a given angle in each cell inside all the blocks of a certain image represent the descriptor of the image. SVM is used to perform the final classification. One of the main drawbacks of the HOG features vector is the high computational cost required for the computation of the features. Modern high efficient techniques using tools such as parallel processing and the reduction of the image to process (only ROIs provided by laser scanner are processed) allow having real time detection.

### 4.3. Fusion Algorithm

The Kalman Filter was considered a robust and reliable choice for tracking pedestrians, thanks to the fast acquisition frequency of the sensor. In [27] a model for a Kalman Filter to track pedestrians using the constant velocity model is given, modeling accelerations as system errors. Equations (5) and (6) present the system error Q and the measurement error covariance matrixes R of the KF for the model used, which models the variations of the velocity of the pedestrian according to the maximum acceleration amplitude (*a_x_*,*a_y_*):

(5) $$Q=\left[\begin{array}{cccc} \frac{{a_{x}}^{2}t^{3}}{3} & \frac{{a_{x}}^{2}t^{2}}{2} & 0 & 0\\ \frac{{a_{x}}^{2}t^{2}}{2} & {a_{x}}^{2} & 0 & 0\\ 0 & 0 & \frac{{a_{y}}^{2}t^{3}}{3} & \frac{{a_{y}}^{2}t^{2}}{2}\\ 0 & 0 & \frac{{a_{y}}^{2}t^{2}}{2} & {a_{y}}^{2} \end{array}\right]$$

(6) $$R=\left(\begin{array}{cc} {\sigma^{2}}_{x} & 0\\ 0 & {\sigma^{2}}_{y} \end{array}\right)$$

where σ^2^_*x*_ y σ^2^_*y*_ is the standard deviation for the measurements in *x*, *y* coordinates. These deviations have been calculated using test sequences (as will be explained in Section 7). As both systems share the ROI coordinates, the deviation in the measurements are considered equal for both detections. The values *a_x_* and *a_y_* in Equation (6) are the maximum amplitude of the acceleration in each axis. According to [28], these amplitudes can be defined as 11 m/s^2^ each.

The constant velocity model for the Kalman filter is defined in Equations (7–10):

(7) $$X=\left[\begin{array}{c} \begin{array}{c} \begin{array}{c} x\\ y \end{array}\\ v_{x} \end{array}\\ v_{y} \end{array}\right]$$

where *X* is the state vector, *x*, *y* represent the location of the pedestrian (in meters) in relation to the vehicle, *v_x_* and *v_y_* are the speed of the pedestrian in meters per second:

(8) $$Y=\left[\begin{array}{c} x\\ y \end{array}\right]$$

*Y* is the measurements vector for the Kalman Filter, with *x*, *y* the location of the new detections, *H* is the observation model matrix and *F* is the state transition model matrix.

(9) $$H=\left[\begin{array}{cc} \begin{array}{cc} 1 & 0 \end{array} & \begin{array}{cc} 0 & 0 \end{array}\\ \begin{array}{cc} 0 & 1 \end{array} & \begin{array}{cc} 0 & 0 \end{array} \end{array}\right]$$

(10) $$F=\left[\begin{array}{cc} \begin{array}{cc} 1 & 0 \end{array} & \begin{array}{cc} t & 0 \end{array}\\ \begin{array}{cc} \begin{array}{c} 0\\ \begin{array}{c} 0\\ 0 \end{array} \end{array} & \begin{array}{c} 1\\ \begin{array}{c} 0\\ 0 \end{array} \end{array} \end{array} & \begin{array}{cc} \begin{array}{c} 0\\ \begin{array}{c} 1\\ 0 \end{array} \end{array} & \begin{array}{c} t\\ \begin{array}{c} 0\\ 1 \end{array} \end{array} \end{array} \end{array}\right]$$

The aforementioned model is used for movement estimation. Several works proved the usability of the constant velocity model to model the movement of pedestrians [28]. Furthermore, the high acquisition rate of the laser scanner (approx. 20 frames per second) which was used as time reference allows a fast adaptation to any speed changes in the movement of the pedestrian.

Data association is the process of combining the new detection with the already existing tracks. The first step is to reduce the possible combination to those obstacle detections located close to the tracks. To do this, a square gate was defined:

(11) $$K_{Gl}\sigma_{r}$$

where *σ_r_* is the residual standard deviation and *K_Gl_* is a constant that was empirically chosen.

After that, association is performed among those detections that are included within the gate of each track, using normalized distance and a stability factor, giving less priority to less stable measurements:

(12) $$d^{2}=\frac{(x_{i}-{\bar{x)}}^{2}}{\sigma_{x}^{2}}+\frac{(y_{i}-{\bar{y)}}^{2}}{\sigma_{y}^{2}}+\ln(\sigma_{x}\sigma_{y})$$

#### Track Management

For track management, the pedestrian definition follows Equation (13):

(13) $$\text{Pedestrian}=\left[\begin{array}{c} X\\ lp\\ cp \end{array}\right]$$

where *X* is the KF state vector defined in Equation (7) and *lp* (laser positive) and *cp* (camera positive) represent the Boolean values that indicate if a pedestrian has been positively detected (1 value) by the corresponding sensor over time. Following this definition, each detection was also defined by Equation (14):

(14) $$\text{Detection}=\left[\begin{array}{c} Y\\ \text{New}\_lp\\ \text{New}\_cp \end{array}\right]$$

As in Equation (13), *New_lp* and *New_cp* provides a positive value (1) or not (0) of the given sensor for a given detection.

Thus after KF update, the Pedestrian state vector is updated with the state vector of the KF and the values of the laser and camera detection are updated with the Boolean sum (OR) of the corresponding values with the detection values in Equation (15):

(15) $$\text{Pedestria}n_{k}=\left[\begin{array}{c} {\hat{X}}_{k|k}\\ \left(lp_{k-1}+\text{New}\_lp_{k}\right)\\ \left(cp_{k-1}+\text{New}\_lp_{k}\right) \end{array}\right]$$

where the state of a given pedestrian in an instant k is defined by the a posteriori state vector provided by the KF *x̂*_(_*_k_*_|_*_k_*_)_ and the Boolean values that indicate a positive detection from the creation of the track by any subsystem.

A track is thus considered consolidated with certainty enough to be reported as a pedestrian, when both *cp* and *lp* provide positive values. The logic followed to track creation and deletion is depicted by the following table:
Track creation-A track is created if a detection does not match with any track.-Every new track is considered consolidated when both sensors detect it.Track deletion-*Not consolidated*. If there is no match after three consecutive scans, the track is deleted.-*Consolidated*. After five no updatesTrack update-Track matching with any of the sensors.

It should be pointed out that Assignment Matrix was used to track association, following at least overall cost assignment [28,29].

## 5. V2V Pedestrian Detection Communication

Once the pedestrians are detected, it is important to alert vehicles in the surroundings, using a fast and reliable V2V communication system. This means that the information about the location of pedestrians and the corresponding warnings alerts have to be sent to other vehicles. Two elements have to be defined from the viewpoint of communications. On the one hand, the communication technology used to support the data transmission that must be adapted to vehicular environments; on the other hand, the structure of the data packages that contain the information to be transmitted from one vehicle to the others. In any case, we consider that each vehicle equips a vehicular communication module and can be considered as a node of the network.

### 5.1. Communication Technology

The communication technology used in the experiments related to this paper is based on Maxfor Inc. MTM-CM3100 gateways, based on the TelosB platform. This allows full connectivity with any PC through a USB port, and is used as interface to access a vehicular mesh network. This device works under the TinyOS open code operating system. In order to access the wireless network to 2.4 GHz in mesh, it uses the IEEE 802.15.4 standard at physical and link level and a mesh geo-networking protocol, which guarantees the desired functionality of the Vehicular Ad-Hoc Network (VANE). This GeoNetworking is supported by a novel GeoNetworking protocol (Geocast Collection Tree Protocol—GCTP) implemented to support the mesh routing. The GeoNetworking protocol is a network protocol that resides in the network and transport layer [30] and is executed in each ad hoc router, specifically in each GeoAdhoc router. This novel protocol reconfigures the network structure in accordance with the GPS positions of the network nodes. With this technology it is possible to develop the necessary algorithm to optimize the message routing in V2V and V2I communications. This algorithm can be used as a solution to be applied to other specifically designed technologies when available.

### 5.2. Data Package Structure

The data package structure contains the format of the information to be transmitted from one communication node to another. It contains the necessary data to support the application of V2V-based pedestrian detection warning.

**Figure 6.**
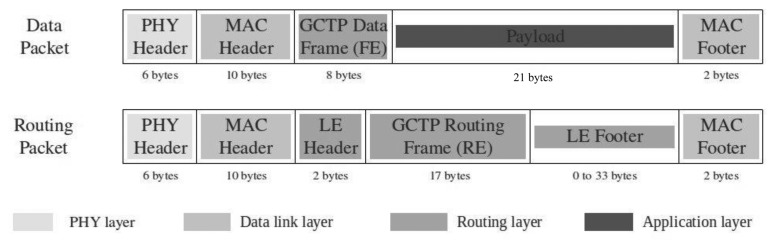
Structure of the data packages to support GeoNetworking and V2V pedestrian detection warning.

Figure 6 shows the structure of both data packets defined to support the application and the GeoNetworking. Thus, the geo-network routing definition uses the routing packets that allow establishing the necessary route tables in mobility to guarantee an efficient information transmission among the nodes of the vehicular mesh network. In this packet the PHY Header field, MAC header, LE Header, LE Footer and MAC Footer represent the standard information of an IEEE 802.15.4 network [31]. The GCTP Routing Frame represents the geo-referenced information used to provide the GCTP algorithm with the necessary information to define the geo-based route tables of the vehicular mesh network.

The data packet schema defines the packet structure to support the application data exchange. Similarly the Routing Packets and the Data Packets are formed by 3 IEEE 802.15.4 standard fields (PHY Header, MAC Header and MAC Footer), a specific Geo-Networking field (GCTP Data Frame) and the Payload that contains the application information.

**Figure 7.**
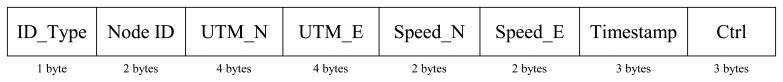
Detail of the Payload of the data packets.

This Payload is described in detail in Figure 7. The different fields that are needed to support the V2V-based pedestrian warning system are shown. The field ID_Type identifies the type of obstacle that is detected by the system, which, in the present application, always corresponds to a pedestrian. The Node ID represents the unique identifier associated with each node of the network. UTM_N and UTM_E represent the GPS Universal Transverse Mercator (UTM) coordinate for the given pedestrian. Speed_N and Speed_E represent the velocity vector of the detected pedestrians, thus all the information related to the status of a given pedestrian, obtained from the KF state, is provided in the payload field. Timestamp is a temporal identifier that represents the instant when the message was generated. Finally, the Ctrl field is a CRC to guarantee the right payload definition.

It is important to remark that to allow real time detection, periodic updating of the detections is mandatory. On the other hand, it is also important to reduce the load of the network. To allow a compromise between both constraints, the updating period of the detection was defined as a parameter in both emitter and receiver, allowing the system to be adapted to reduce the workload without losing real time performance.

### 5.3. Data Transmission Operation

The nodes of the network link one to another at a maximum distance of 100 m. The link means that the GeoNetwork is established but no data transmission is required. The data transmission begins in the moment that the pedestrian detection system detects a pedestrian on the route of the vehicle. In this moment, the system starts to broadcast the pedestrian warning through V2V communications. The receptor vehicles retrieve the data frames and their information and decide whether to warn the driver, depending whether or not they are in the influence area of the receiver.

## 6. Test & Results

Several tests were performed, divided into three sets. First, a calibration test was performed to tune up the KF (Equation (6)) and to check the accuracy of the obstacle detection algorithm. In the second test, validation tests were performed, where the entire algorithm was tested in a controlled environment obtaining matching results for each sensor performance independently and for the complete system. In this tests set, performance of the system was checked, as well as the parameters of the application, which was varied to obtain the best configuration (e.g., number of negative detections to eliminate a track) to find the final configuration. Finally, tests in real road situations were performed where false positives are more common due to the variety of pedestrians and other unpredicted obstacles.

### 6.1. Calibration Test

**Figure 8.**
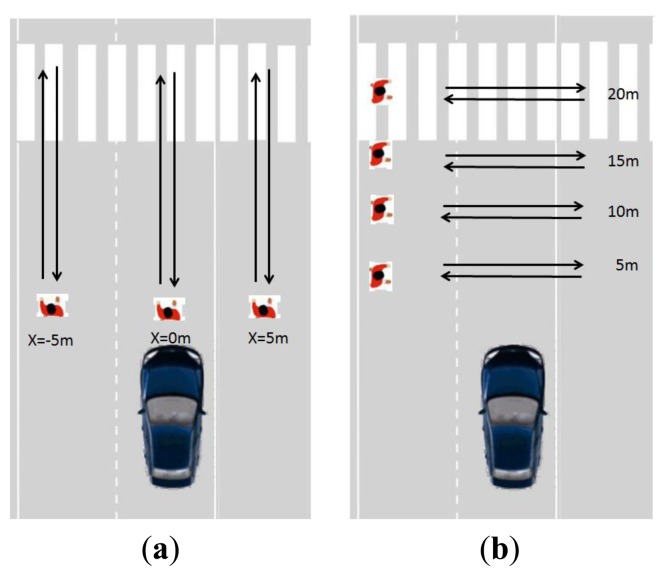
The experiments aimed at measuring the measurement errors. (**a**) x position is fixed. (**b**) y position is fixed.

This test consisted of test sequences with a single pedestrian performing lateral and vertical movements. In lateral movements the y coordinate was fixed, so the system could measure the error in the y coordinate as the pedestrian moves along the x axes. For vertical movements, the pedestrian had the x coordinate fixed and moved along the y axis, thus the deviation in x was measured (Figure 8). Furthermore, this test helped to check the performance of the laser scanner detection and segmentation algorithm and the accuracy to the estimation of the movement of the pedestrian. The tests were performed over 20 pedestrians, all performing the same movements, in controlled scenarios. The movements were predefined, providing ground truth, used to check the accuracy of both laser scanner detection and KF estimation. Subsequent tests performed in real road scenarios were used to check the reliability of the detection algorithm.

**Figure 9.**
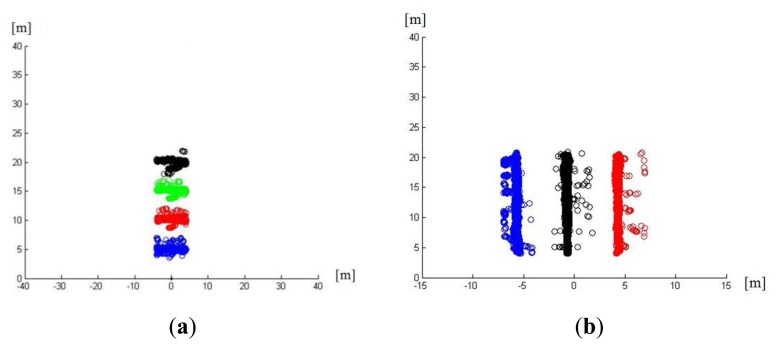
Results of the experiments to measure the measurement errors y (in meters) *vs.* x (in meters). (**a**) Total measurement of the experiments for lateral movement. (**b**) total measurement of the experiments for vertical movement. (**c**) y error *vs.* x coordinates in lateral experiments. (**d**) x error *vs.* y coordinate in vertical movement experiments.

The results of these test sequences (presented in Figure 9) showed that the assumption of two independent errors for both measurements (x,y) was correct since the error pattern is similar among the lateral movements and vertical movements—regardless of the values on the other axis. Only lateral movements present a higher error when the pedestrian is in the center that was negligible for the present application.

Finally, static axis, allowed checking accuracy of the obstacle detection and tracking, providing a standard deviation of 0.15 m in each axis for laser scanner localization and 0.2 m of mean error for KF estimation. This accuracy allows a trustable detection system able to provide obstacle location with high precision.

### 6.2. Validation Tests

**Figure 10.**
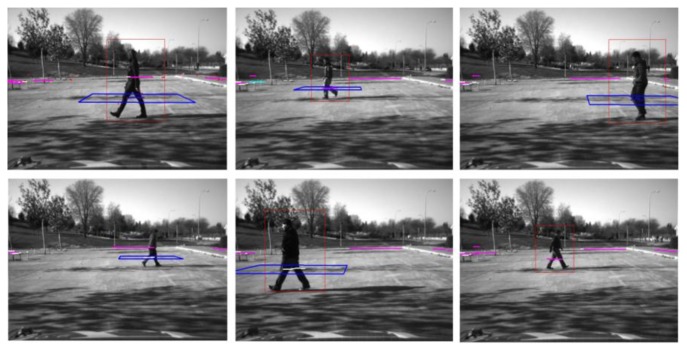
Several validation tests results. Red squares stand for positive detections of the camera. Blue squares constitute laser scanner detections. In the image polyline projections in the image space are also represented.

Validation tests were performed in a parking lot with a single pedestrian simulating pedestrian crossing (Figure 10). Up to nine different pedestrians were tested (Figure 10) when the vehicle was both in movement and stopped. Detections results were 86.2% for computer vision approach, 90.63% for pedestrian approaches and 96.02% for the Fusion system. Here some details need to be remarked on to clarify those results.

- The tests were performed using the best scenario possible. False positives were at a minimum due to the fact that the parking lot represented a controlled scenario where pedestrians were easy to detect, thanks to the absence of other obstacles that could make the detections difficult because of the similarity to pedestrian patterns or occlusions. So false positives found in this test were negligible.
- Laser scanner detection has a higher rate than vision scanner. That could lead to the wrong assumption that the system developed for laser scanner detection is more robust and trustable. As explained in the laser scanner detection subsystem, the limited information provided by the sensor makes classification a challenge, as false positives are common, thereby decreasing the reliability of the detection system. For the present test, as the controlled scenario presented no other obstacles, no false positives were found but it should be taken into account in real scenario tests.
- It is also proved that the detection performance is clearly increased by the fusion approach presented in this paper.

### 6.3. Real Situation Tests

**Figure 11.**
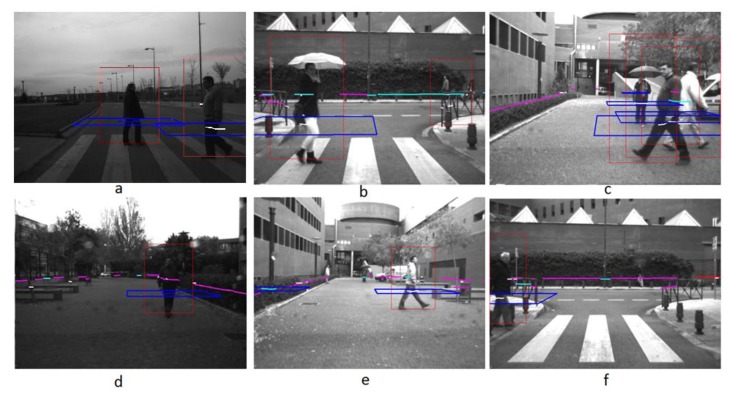
Several detections with subsystems detection highlighted. (**a**) and (**b**) show two detections. (**c**) shows three pedestrians crossing. (**d**), (**e**) and (**f**) show single positive detection.

**Figure 12.**
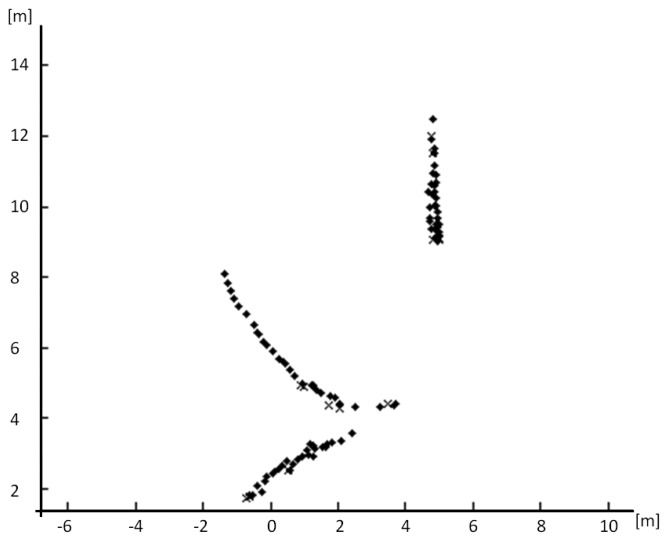
Results for the tracking of the pedestrians in Figure 11c, in which three pedestrians are tracked. Dots represent normal detections. Crosses represent detections where the track is not updated. The vehicle was moving at 20 km/h and stops before the pedestrian.

These tests included several pedestrians walking in real road situations including a single pedestrian crossing (Figure 11b, d, e and f) and several pedestrians in crossing trajectories (Figure 11a, c). Test performed in real roads include both urban and interurban scenarios. The later represents easier scenarios for pedestrian detections regarding to the absence of other obstacles that could lead to misdetection. Urban scenarios represent more challenging scenarios with higher diversity of obstacles, thus misdetections are expected, although the velocities of the vehicles are lower. Figure 12 shows, as example, the entire tracking for Figure 11c, with several pedestrians involved, in different directions, including a static pedestrian. Here, the algorithm is able to track all the pedestrians even *in situ* ations where the pedestrians are crossing, with several occlusions. It is also noticeable that the standing pedestrian is detected and tracked from the beginning of the sequence. The high performance of the system is proved in the numerous sequences tested, as depicted in Table 1.

In the scope of these tests, the real time performance of the system was tested. The results showed that, by means of the parallelization techniques, modern processors, and by reducing the regions on the images where the visual algorithm searches, real time performance is achieved, adding no delays in the detections. Thus the response time is mainly limited by the acquisition frequency of the sensors. As commented before, the high acquisition frequency of the laser scanner allows it to detect pedestrians with a rate of approximately 20 Hz (56 milliseconds). At high speed (120 km/h) it represents less than 2 m covered by the vehicle in each scan, providing real time performance. Thus the limitations of the system presented are linked to the limitations inherent to the sensors, but not related to the work presented here (*i.e.*, the laser scanner detection distance, or computer camera field of view).

### 6.4. Communication Tests

**Figure 13.**
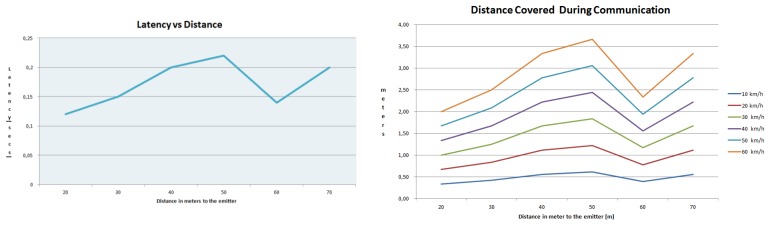
Network performance. Left, latency results *vs.* distance to the emitter. Distance covered by the receiver, according to the velocity and the distance to the emitter (given the latency of the communication).

After checking the performance of the detection algorithm, the network performance was tested to prove the viability of a VANET to alert vehicles in the surroundings, when a pedestrian is detected. The test to measure the latency in the communication process was performed. This test checked the time necessary to send the necessary data to alert another vehicle of a given detection. The performance of the network guarantees the effectiveness of the assistance system, maintaining the average latency lower than 0.17 s, proved in a set of tests at several distances in the operating range. The average behavior of the network is demonstrated in Figure 13.

**Figure 14.**
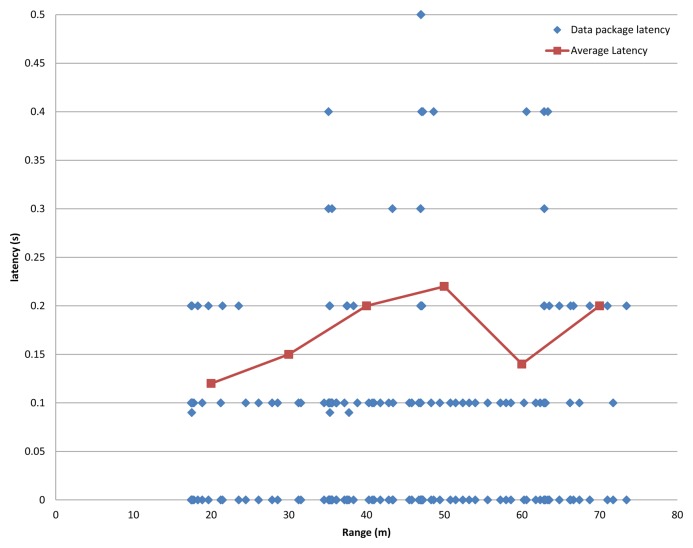
Results of the performance tests of the mesh GeoNetwork that supports the assistance system.

Finally, in Figure 14, the performance of the network in these latency tests is shown graphically, combining several distances, at a data packets transmission rate of 4 Hz during 384 s, obtaining a data loss rate of 0.2%.

This test proved that the performance of the communication protocol is very efficient and effective for safety application with a loss rate of approximately 0.2%, proving the high reliability of the system. Besides, the test performed showed a fast response able to provide alarm communication within a short distance covered by the vehicle, as shown in Figure 13.

## 7. Conclusions

The paper presented provides a step forward in the following aspects:

- An improved laser scanner approach that provides pedestrian detection with the limited information provided by the laser scanner, by means of a pattern matching algorithm augmented with tracking stage and context information.
- Fusion algorithm, able to combine detections from the laser scanner and computer vision approach at a high level.
- An efficient and reliable VANET protocol able to provide information of the detected pedestrians to the vehicles in the surroundings with time enough in advance.

Tests under real conditions proved the viability of the algorithms and the performance of the system in challenging scenarios. The viability of the laser scanner for pedestrian detection was proved (up to 92%). Although a lot of work must still be performed in order to reduce the amount of false positives to values suitable for a single sensor safety application, the laser scanner overall performance was proved to be very useful when combined with the camera detections.

The novel fusion approach for pedestrian detection has proved that, by adding laser scanner pedestrian detection and context information, performance and trustability can be increased, fulfilling the demanding requirements required for traffic safety applications. Furthermore the tracking stage presented was able to track the different pedestrians in variable urban scenarios with high accuracy and trustability.

On the other hand, the efficient V2V communication protocol proved to be able to alert surrounding vehicles even at distances up to 100 m with time in advance to warn the drivers of the dangerous situation, allowing avoiding accidents caused by pedestrian occlusions. The present work has proved that, by means of an effective data fusion algorithm, and a communication protocol, it is possible to create a powerful tool able to increase road safety. This represents a step forward, benefiting from the advantages of the state-of-the-art sensors available in order to provide a novel application able to give multimodal detection.

## Figures and Tables

**Table 1.** Results of the overall system with low level comparison.

	**Camera**		**Laser Scanner**		**Fusion**	

	**%Positive**	**%False Positives**	**%Positive**	**%False Positives**	**%Positive**	**%False Positives**
**Test**	86,2	5.19	90,63	16.23	**96.02**	**0.89**
**Real Road**	71.57	7.39	89.94	16,84	**92.85**	**4.24**
